# Silicene nanomeshes: bandgap opening by bond symmetry breaking and uniaxial strain

**DOI:** 10.1038/srep20971

**Published:** 2016-02-10

**Authors:** Tian-Tian Jia, Xin-Yu Fan, Meng-Meng Zheng, Gang Chen

**Affiliations:** 1 Laboratory of Advanced Materials Physics and Nanodevices, School of Physics and Technology, University of Jinan, Jinan, Shandong 250022, P. R. China; 2 Shandong Provincial Key Laboratory of Laser Polarization and Information Technology and Department of Physics, Qufu Normal University, Qufu, Shandong 273165, P. R. China

## Abstract

Based on the first-principles calculations, we have investigated in detail the bandgap opening of silicene nanomeshes. Different to the mechanism of bandgap opening induced by the sublattice equivalence breaking, the method of degenerate perturbation through breaking the bond symmetry could split the π-like bands in the inversion symmetry preserved silicene nanomeshes, resulting into the π_a1_ − π_a2_ and π_z1_ − π_z2_ band sets with sizable energy intervals. Besides the bandgap opening in the nanomeshes with Dirac point being folded to Γ point, the split energy intervals are however apart away from Fermi level to leave the semimetal nature unchanged for the other nanomeshes with Dirac points located at opposite sides of Γ point as opposite pseudo spin wave valleys. A mass bandgap could be then opened at the aid of uniaxial strain to transfer the nanomesh to be semiconducting, whose width could be continuously enlarged until reaching its maximum E_max_. Moreover, the E_max_ could also be tuned by controlling the defect density in silicene nanomeshes. These studies could contribute to the understanding of the bandgap engineering of silicene-based nanomaterials to call for further investigations on both theory and experiment.

Silicene as a counterpart of graphene has recently gained explosive studies due to its many amazing unique properties such as ultra-high carrier mobility, anomalous quantum Hall effect, and topological insulating state^1^,^2^,^3^. Unlike the ideal flat graphene, the silicene consists of two separate sublattices forming the low-bulked geometrical structure owe to the mixing of *sp*^2^ and *sp*^3^ hybridizations, which provide possibilities in tailoring properties by surface functionalization *etc.* An advantage of silicene over graphene is that it would be easily integrated into the high-performance nanoelectronics based on the current Si-based semiconductor industrial techniques. The silicene has been successfully fabricated on many supporting surfaces, such as the Ag(111)^4^,^5^, ZrB_2_(0001)^6^, Au(110)^7^, and Ir(111) surfaces^8^. Recent studies show also the possibilities in fabricating quasi free standing silicene on non-metallic substrates^9^,^10^,^11^,^12^. These progresses have paved the way for studying its peculiar properties and potential applications. Besides the studies on the synthesis of silicene, the structural defect in silicene has also attracted much attention in view of the property modulation^13^,^14^,^15^,^16^,^17^,^18^,^19^,^20^,^21^,^22^. Several theoretical studies have been devoted on investigating the geometrical structures and modification on silicene’s properties of single and double vacancy defects. For example, the self-healing of vacancy defects in silicene has been found to be driven by the energy gain through new Si-Si bond formation^13^. Guo *et al*.^14^ studied in detail the structure, mobility, electronic and magnetic properties of vacancy defect in silicene. Şahin *et al*.^15^ have evaluated the formation, stability, and reactivity of defect. In Berdiyorov *et al*.’s studies^16^, the effects of vacancy defect on the thermal stability of silicene has been investigated with molecular dynamics method. Interestingly, a recent experimental study^17^ has shown the presence of a large number of defect clusters which has recently been carefully investigated by Li *et al*.^18^ with *ab initio* molecular dynamics simulations.

Considering the experimental advances in patterning two-dimensional materials by regularly arranging vacancy antidots to fabricate nanomeshes^23^, the effects of vacancy hole on bandgap opening in silicene has been analyzed in detail^19^,^20^,^21^,^22^. A bandgap engineering mechanism–degenerate perturbation which is different to the sublattice equivalence breaking has been proposed. In fact, this phenomena could be seen in a recent experimental study on the fabricating of silicene on ZrB_2_(0001) surface^6^, where the periodic pattern of interaction between silicene and substrate modulates the boundary conditions of silicene. The silicene was actually patterned into superlattice with the Dirac cone being folded to Γ point and a bandgap was confirmed to be opened at Γ. The bandgap opening is a critical and attractive research issue for silicene to be used in photoelectronics, where a proper on/off ratio is required. The ideal free-standing silicene, though it has high velocity massless Fermions as charge carrier owe to the nearly linear energy dispersion relation around Dirac cone, has to be transferred from semimetal to semiconductor to be used for transistors in nanoelectronics. A kind of nanomeshes with Dirac cone being folded to Γ to form four-fold degeneracy could be opened a bandgap by the degenerate perturbation through introducing an inversion symmetry preserved antidot in the repeated unit. Such antidot would however not change the semimetal nature of the other nanomeshes which are still less of investigations. Actually, the π-like band splitting also happens in such nanomeshes, which is however missed in the previous studies^19^,^20^,^21^,^22^. In this paper, we would show that such band splitting can also transfer the nanomesh to be semiconducting at the aid of strain.

## Results

**Figure 1:**
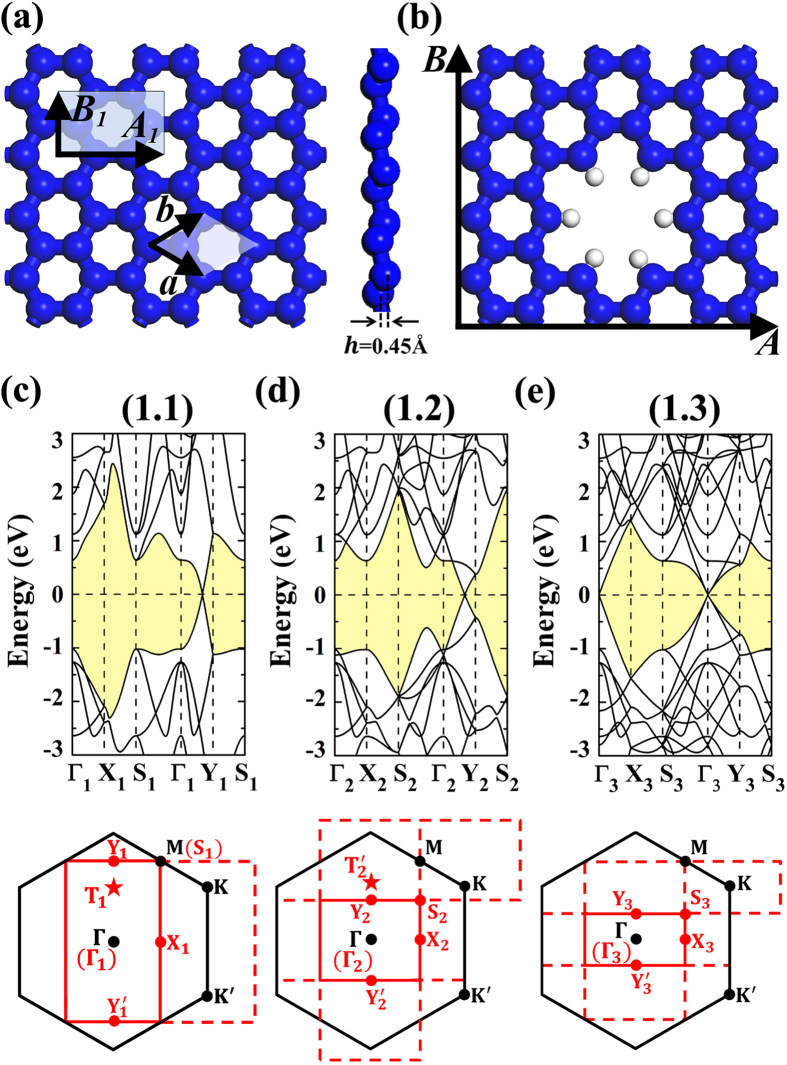
The top and side views of silicene structure (**a**) and the schematic geometry of the nanomesh ***A*** × ***B*** patterned by regularly arranged antidots (**b**). The big and small balls stand for the silicon and hydrogen atoms, respectively. In (**a**), besides the ***a*** × ***b*** primitive unit cell, the smallest rectangle unit ***A***_1_ × ***B***_1_ is also shown. In (**c**–**e**), the upper and lower panels are for the bandstructures and Brillouin zones, respectively. Besides the *r*-BZ (red rectangle) for each PSS, the *h*-BZ (black hexagon) for the primitive cell is also presented for band folding analysis.

Both the hexagonal and orthogonal silicene nanomeshes could be fabricated in experiment. In this paper, we would like to concentrate on studying the orthogonal nanomeshes to facilitate the discussion of the uniaxial strains respectively applied along armchair and zigzag directions, whose conclusions could shed light on the hexagonal nanomeshes accordingly. In Fig. 1, besides the primitive unit ***a*** × ***b*** of silicene, the smallest orthogonal unit ***A***_1_ × ***B***_1_ with ***A***_1_ = ***a*** + ***b*** and ***B***_1_ = -***a*** + ***b*** has also been schematically illustrated. The repeated unit shown in Fig. 1b for the antidot patterned nanomesh could be written as ***A*** = *M**A***_1_ and ***B*** = *N**B***_1_, which could be hereafter accounted by (*M*,*N*). In order to facilitate discussion, we would like to refer the defect free silicene calculated by using the (*M*,*N*) supercell as pseudo silicene superlattice (PSS). Therefore, the (1,1) PSS stands for the pristine silicene calculated with the smallest rectangle unit ***A***_1_ × ***B***_1_, whose bandstructure has been shown in Fig. 1c. The Dirac point is found between Γ_1_ and Y_1_. The lower panel of Fig. 1c shows the rectangular Brillouin zone (*r*-BZ) of (1,1) PSS and the hexagonal Brillouin zone (*h*-BZ) corresponding to the primitive unit cell. One could see that the K (K′) in the *h*-BZ is now equivalent to the 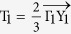 (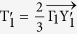) in the *r*-BZ, agreeing with the Dirac point position in the calculated bandstructure. Similar analysis on the (1,2) PSS shows the band folding of K (K′) point Dirac cone in *h*-BZ of primitive cell to the 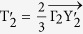 (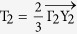) point in *r*-BZ of (1,2) PSS. The corresponding bandstructure and band folding analysis are presented in Fig. 1d. The results shown in Fig. 1e for the (1,3) PSS show that its Dirac cone is folded to Γ point. Based on the detailed band folding analysis, we would like to sort the studied orthogonal lattices into three categories depending on the band folding of Dirac cone: (1) the 3*p* PSS (*M*,*N*) with *N* = 3*p*, folding to the Γ in the corresponding *r*-BZ; (2) 3*p* + 1 PSS with *N* = 3*p* + 1, folding to the 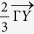; and (3) 3*p* + 2 PSS with *N* = 3*p* + 2, folding to the 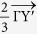. Compared to the 3*p* type superlattices, the 3*p* + 1 and 3*p* + 2 type superlattices are less of studies till now.

**Figure 2:**
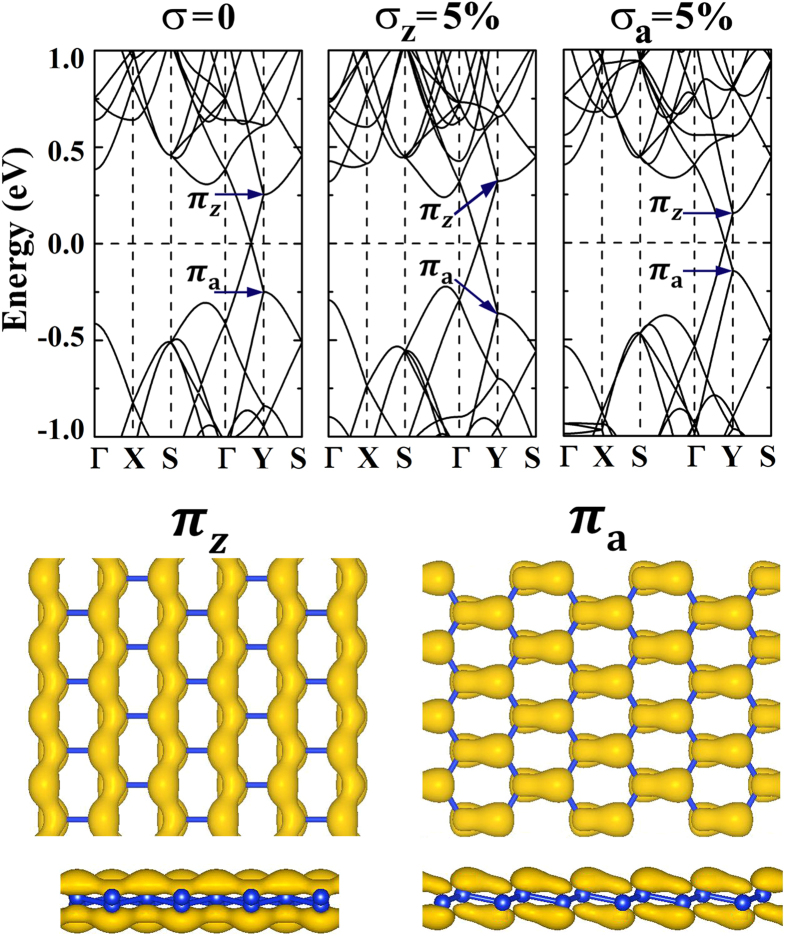
The banstructure of the free-standing (3,4) PSS and those under σ_z_ = 5% and σ_a_ = 5% unaxial strains. For the free-standing (3,4) PSS, the band decomposed charge densities for the bands along ΓY path as marked by π_z_ and π_a_ are studied at Y point with the iso-value of 0.01 e/Å^3^. Both top and side views are shown.

We would like to start our studies by showing the strain effects on the 3*p* + 1 and 3*p* + 2 type PSSes. For the orthogonal lattice (*M*,*N*), the strain could be respectively applied along the armchair (***A*** lattice) and zigzag (***B*** lattice) directions, which we would like to refer as σ_a_ and σ_z_. In Fig. 2, the (3,4) PSS is adopt as prototype to illustrate our results on the 3*p* + 1 lattices. The uniaxial strain would continuously shift the Dirac cone along the 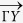 path. The σ_a_ would shift it toward Y point while the σ_z_ would move it close to Γ. The effects of the strain could also be accounted by the energy interval ΔE between the π_z_ and π_a_ as marked in the Fig. 2. As to the marked bands, we have also calculated their band decomposed charge densities. The corresponding densities of the isolated (3,4) PSS are presented in Fig. 2, which suggest the π-like bonding characters. Also, considering the fact that the electronic configuration of silicene could be regarded as the mixture of *sp*^2^ and *sp*^3^ hybrizations, we would like hereafter to discuss them as π-like bands. The densities of the π_a_ and π_z_ bands are found to be respectively distributed around the bonds along armchair and zigzag directions. As shown in Fig. 2, the ΔE would be enlarged (reduced) by the σ_z_ (σ_a_) strain. Our careful analysis shows that similar strain effects could also be found in the 3*p* + 2 PSSes. The difference is that the ΔE would be reduced (enlarged) by the σ_z_ (σ_a_) strain, respectively.

**Figure 3:**
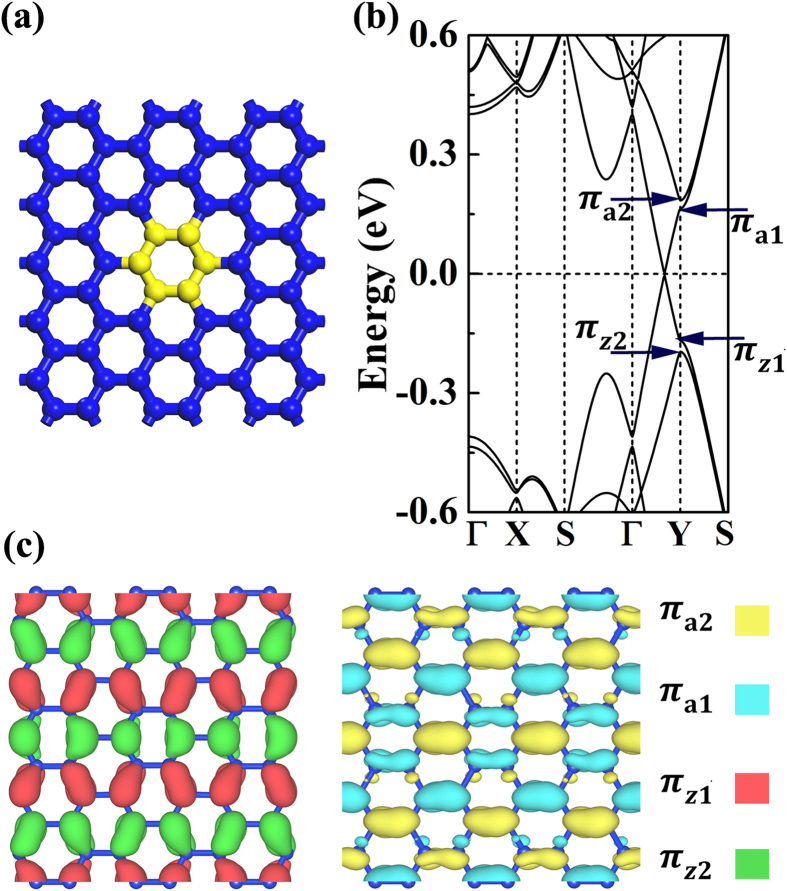
The schematic structure of the ISP defect introduced by contracting the highlighted silicon hexagon (**a**) and the corresponding bandstructure (**b**). The π-like band splitting at Y point is marked out for eye guidance. The split bands along ΓY path are marked by π_a1_, π_a2_, π_z1_, and π_z2_ at Y point. Their band decomposed charge densities at Y point are studied in (**c**) with the iso-value of 0.01 e/Å^3^.

For the 3*p* + 1 and 3*p* + 2 type silicene superlattices, the defect can also induce band splitting phenomena. The inversion symmetry preserved (ISP) defect has been found to open bandgap in the 3*p* silicene nanomeshes, which would however not alter the conducting properties of the 3*p* + 1 and 3*p* + 2 nanomeshes^19^,^20^,^21^,^22^. The periodically arranged defects would impose new Born von Karman boundary conditions on silience to make it into superlattice^24^,^25^, which have been introduced in by the modulation of the Bravais lattice. In the defect free pristine silicene crystal, the hexagonal primitive unit cell defined by ***a*** × ***b*** (see Fig. 1) accounts for the Bravais lattice of silicene, which should correspond to the ***A*** × ***B*** periodic unit in the (*M*,*N*) superlattice. The periodically arranged defects would alter the Bravais translation symmetry to modulate the Born von Karman boundary conditions accordingly. In the ISP defect patterned superlattice, besides the previously discussed band splitting in 3*p* type superlattices at Γ point to open bandgap, the ISP defect in fact would also induce band splitting phenomena in the other superlattices. In order to facilitate discussion, we would like to mimic an ISP defect in silicene by contracting a silicon hexagon by about 3%, which is highlighted in Fig. 3a. In our simulations, the atoms in this hexagon have been fixed while the rest atoms are free to relax. The methodology of tight-binding method^24^ is believed to be efficient in dealing with silicene which could help to understand the physical origin of the electronic properties of silicene-based nanostructures. By considering only the nearest neighbor atoms, the related energy E_nna_ could be obtained through


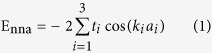



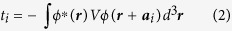


where *t*_*i*_ is the transfer integral, *k*_*i*_ is the component of wavevector ***k**, a*_i_ is the corresponding lattice constant, and 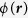 is the atomic orbital. According to the TB method, the ~3% contraction of the Si-Si bonds in the highlighted silicon hexagon would enhance the corresponding transfer integrals to introduce perturbation to the electronic properties of silicene. Such periodically arranged perturbation would then make the silicene superlattice. In Fig. 3, in such ISP defect patterned (3,4) superlattice, the π_z_ and π_a_ bands at Y point are found to split into π_z1_, π_z2_, π_a1_, and π_a2_ bands. The bandgap E_z_ of the π_z1_ − π_z2_ band set and the one E_a_ of the π_a1_ − π_a2_ band set equal to each other. Both of them are however apart away from the Fermi level leaving the semimetal nature unchanged. In our band decomposed charge density studies of the split π-like bands at Y point, the π_z1_, π_z2_, π_a1_, and π_a2_ bands are found to correspond to four different bond groups as schematically illustrated in Fig. 3c.

## Discussion

In the ideal free-standing silicene sheet, the *sp*^2^ and *sp*^3^ hybridization electronic configuration makes the Si-Si bonds in silicene to be same, whose π-like electrons would be thus high-symmetrically distributed along the bonds in the hexagonal honeycomb lattice. Therefore, the π-like bands are degenerate. By applying uniaxial strain on the silicene, the equivalence among the Si-Si bonds would be broken. As to the cases studied in Fig. 2, the 5% σ_z_ strain would make the π_z_ bonds to elongate of 2.1% while the π_a_ bonds only slightly contracts (~0.2%). The σ_a_ strain would induce the 3.0% elongation of π_a_ bonds and 0.7% change of π_z_ bonds. The different bond length changes along π_a_ and π_z_ bonds would result in different hopping integrals, which would then perturb the 3-fold homogeneously distributed electronic structure to split the π-like bands. The slight contraction (elongation) of bond length would induce the enhancing (weakening) of the binding strength of the band electrons to split the degenerate π-like bands, which stands for the increase and decrease of band energy interval of π_a_ and π_z_ bands presented in Fig. 2.

As studied in Fig. 3, by introducing a Si_6_ ISP defect into the repeated unit to pattern (3,4) silicene superlattice, the π-like bands are found to be split due to the defect induced perturbation. After contracting the Si_6_ hexagon to generate the ISP defect, the equivalence among the 3-fold homogeneously distributed Si-Si bonds is broken. In our studies, the π_z1_, π_z2_, π_a1_, and π_a2_ bands are calculated to gain 0.006, −0.011, −0.014, and 0.007 Å bond elongation on average as compared to the corresponding bond lengths of the defect free (3,4) PSS. According to the tight-binding method^24^, the bond elongation (contraction) would induce the decrease (increase) of the corresponding transfer integral. As a result, the π-like band splitting happens. The π_a1_ and π_z2_ bands are found to have lower band energies as compared to the π_a2_ and π_z1_ bands, respectively. The π band splitting was previously discussed in the 3*p* type superlattices which could be opened a mass bandgap to transfer the superlattices into semiconducting materials^19^,^20^,^21^,^22^. The intervalley scattering was proposed to understand the band gap opening. The periodically arranged defects could be approximately treated as introducing a periodic potential V_d_(***r***) to the pristine silicene. If the wavervectors of the opposite ***D*** and ***D***^′^ valleys (the positions of the opposite Dirac cones) differ with each other by a reciprocal lattice ***G*** or mach with each other at a same point, the intervalley scattering would induce band splitting whose split energy interval E_d_ would be proportional to the perturbation related integration^20^.


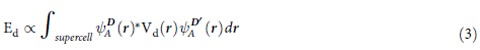


where 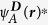 and 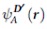 are the Bloch functions of sublattice A at the opposite valley points ***D*** and ***D***^′^. In the 3*p* type superlattices, such understanding of the band splitting could hold. However, in the 3*p* + 1 and 3*p* + 2 type superlattices, the wavevector between two nearest opposite Dirac points is 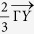 failing to satisfy the above mentioned conditions. Providing we could talk about the 3-fold homogeneous equivalence of Si-Si bonds in silicene as bond symmetry, the band splittings happened in the strained defect-free silicene and the free-standing defect patterned superlattices may be then attributed to the bond symmetry breaking. Here, we would like to conclude the main contribution of our studies to the knowledge of bandstructure engineering of the two dimensional materials as compared to the previous studies^19^,^20^,^21^,^22^. We have carefully investigated the band splitting phenomenon of the π-like bands of the 3*p* + 1 and 3*p* + 2 type superlattices, which was missed in the previous studies. Furthermore, the corresponding band splitting mechanism could not be understood by the previously proposed intervalley scattering, which could be attributed to the bond symmetry breaking based on our detailed analysis. The split intervals are however apart away from the Fermi level to leave the semimetal nature unchanged. Fortunately, as to be discussed later in this paper, such energy intervals could be manipulated to approach each other to open a mass bandgap to transfer the silicene nanomesh to be semiconducting at the aid of uniaxial strain.

**Figure 4:**
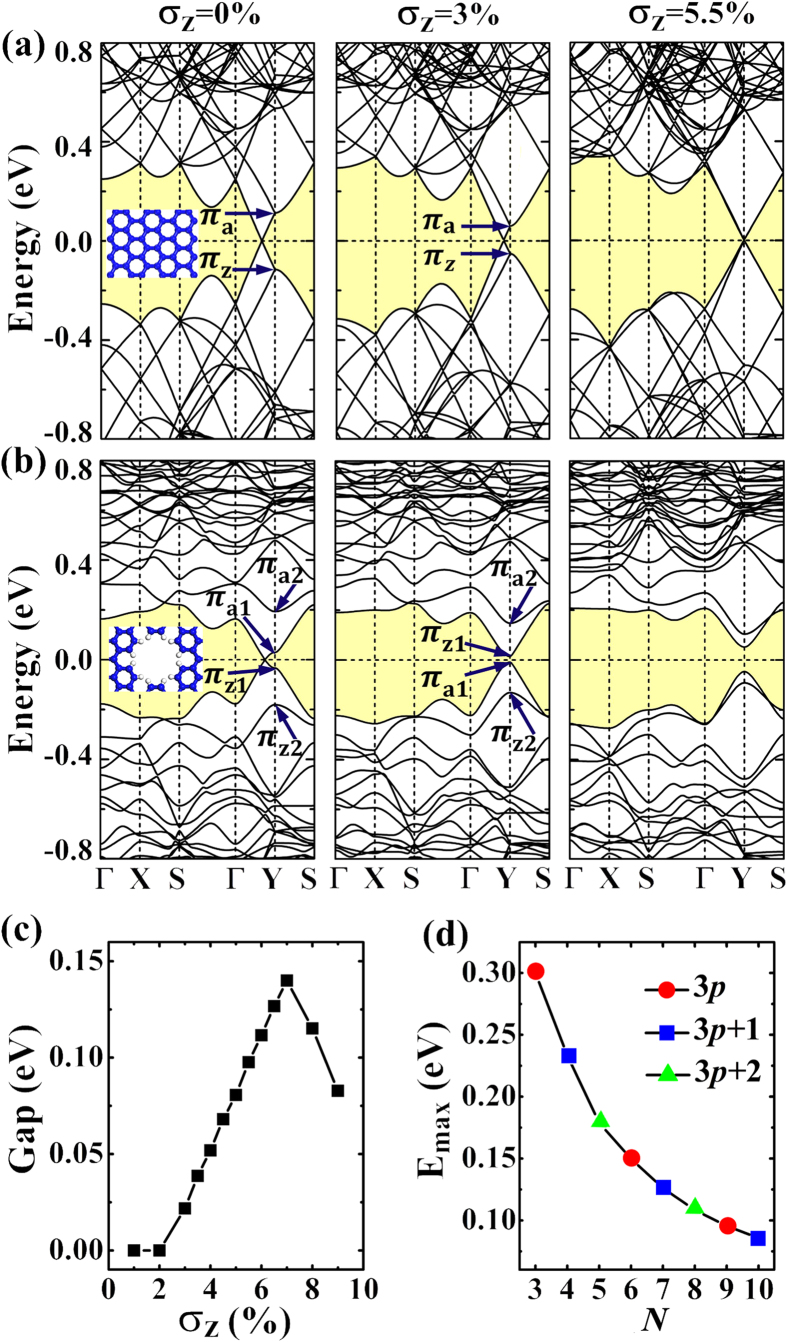
The bandstructures of the free-standing (6,8) PSS and the ones under 3%, 5.5% σ_z_ strains (**a**) and the corresponding bandstructures for the Si_12_ antidot patterned (6,8) nanomeshes (**b**). The evolution of the mass bandgap as a function of the applied strain for the (6,8) nanomesh (**c**) and the relationship between E_max_ and lattice size *N* of (*6*,*N*) nanomeshes (**d**). The bandgaps of 3*p*-type nanomeshes are also presented in (**d**) for comparison.

Now, we discuss the combination effects of strain and defect on bandstructure engineering. To shed light on the experimentally synthesized nanomeshes patterned by regularly arranged antidots, the Si_12_ antidot patterned silicene nanomeshes are examined as prototype in our studies, which corresponds to the vacancy hole formed after removing the Si_12_ nanoflake from silicene sheet. (In order to facilitate discussion, we would like to use the notation Si_n_ to account for the antidot formed by removing a *n*-atom *D*_6h_ silicon nanoflake from the silicene with the hole edge passivated by hydrogen.). Considering the reactivity of the dangling bonds, the vacancy hole edge would be ready for functional group adsorption in experimental studies. In this paper, the hydrogen atoms are used to saturate the dangling bonds to mimic the real nanomeshes to shed light on the experimental studies. After the hole edge hydrogenation, the silicene nanomeshes have been found to be nonmagnetic in our calculations. As to the silicene superlattices patterned by the antidots without dangling bond passivation, the resulted materials would be probably magnetic due to the presence of the unpaired π-like electrons on the hole edge, which have already been confirmed in the antidot studies of graphene^21^. The ferromagnetic coupling could keep the inversion symmetry while the antiferromagnetic one would break it, which are worthy of detailed studies. However, such studies are beyond our scope of this paper to understand the band splitting mechanism of the 3*p* + 1 and 3*p* + 2 nanomeshes. In Fig. 4, we use the (6,8) lattice as an example to illustrate our results. In Fig. 4a, the effects of σ_z_ strain on the (6,8) PSS are studied, which would shift the Dirac point toward Y point. At the strain of σ_z_ = 5.5%, the Dirac cone is found at the Y point. As to the Si_12_ antidot patterned (6,8) nanomesh studied in Fig. 4b, the π-like band splitting happens. The applied uniaxial strain σ_z_ would continuously lower the π_a1_ − π_a2_ band set toward the Fermi level, while it would lift the π_z1_ − π_z2_ band set. Once the E_a_ and E_z_ start to overlap, a mass bandgap straddle over Fermi level would start to form to transfer the nanomesh from semimetal to semiconductor. As presented in Fig. 4c, the critical value of uniaxial strain for such transition is found to be around 2% for the Si_12_ antidot patterned (6,8) nanomesh.

Furthermore, the mass bandgap could be continuously enlarged by enhancing strain until reaching its maximum E_max_ which equals to E_a_ (or E_z_). Upon this point, it would get narrower when the strain σ_z_ further increases (see Fig. 4c). Based on our detailed calculations, we would like to conclude that the mass bandgap opening and the width tuning of such bandgap can be realized by applying uniaixal strain on all the 3*p* + 1 and 3*p* + 2 type (*M*,*N*) silicene nanomeshes. In our studies, we have also investigated the possibilities in tuning the E_max_. The (6,*N*) nanomeshes are carefully studied. As shown in Fig. 4d, the E_max_ would reduce along with the increase of the lattice size *N* to reduce the density of the defect in silicene nanomesh.

In conclusion, we have carefully studied the silicene nanomeshes. Besides bandgap opening in the 3*p* nanomeshes which are transferred to be semiconducting due to the four-fold degeneracy removing, the inversion symmetry preserved defect can also induce band splitting in the other nanomeshes. However, the split gaps of π_a_-type and π_z_-type bands are apart away from the Fermi level leaving the semimetal nature unchanged, which fortunately could be shifted toward each other by applying uniaxial strain. Once they start to overlap, a mass bandgap would appear to transfer the nanomesh to be semiconducting, which could also be continuously enlarged until reaching its maximum E_max_. Furthermore, the E_max_ could be tuned by controlling the defect density in silicene nanomesh.

## Methods

We have performed first-principles calculations with the spin-polarized density functional theory by using the Vienna *ab initio* simulation package^26^. The interaction between ion and electron was described by the projector augmented-wave method^27^. The generalized gradient approximation with the Perdew, Burke, and Ernzerhof^28^ parameterized functional was used to account for the exchange-correlation energy among electrons. The wavefunction was constructed with the planewave basis set with the 250 and 400 eV cutoff energies for respectively dealing with the undoped pristine slicene-based nanostructures and the silicene nanomeshes patterned by the periodically arranged antidots. The method of the supercell was employed to simulate the two dimensional material by placing the sheet crystal in the XY plane which is separated by about 15 Å vacuum along the perpendicular direction to eliminate the interaction with its neighboring images. The convergence tolerances were 10^−5^ eV for the total energy and 0.01 eV/Å for the Feynman force acting on each aotm. The integration of electronic properties was calculated by using the Monkhorst-Pack technique^29^ to sample the Brillouin zone. Due to the small sizes, the structures studied with the primitive unit cell and the smallest rectangle unit cell were calculated with the 21 × 21 × 1 *k*-mesh. As to the orthogonal superlattices investigated in our studies, the sizes of the repeated units are 20.1 × 11.6 × 15 Å^3^ and 40.2 × 23.2 × 15 Å^3^, respectively. Accordingly, the *k*-meshes of 5 × 7 × 1 and 3 × 5 × 1 were used for studying their electronic properties.

## Additional Information

**How to cite this article**: Jia, T.-T. *et al*. Silicene nanomeshes: bandgap opening by bond symmetry breaking and uniaxial strain. *Sci. Rep.* **6**, 20971; doi: 10.1038/srep20971 (2016).
